# Implementing Impact Evaluations of Malaria Control Interventions: Process, Lessons Learned, and Recommendations

**DOI:** 10.4269/ajtmh.17-0064

**Published:** 2017-09-27

**Authors:** Christine L. Hershey, Achuyt Bhattarai, Lia S. Florey, Peter D. McElroy, Carrie F. Nielsen, Yazoume Yé, Erin Eckert, Ana Cláudia Franca-Koh, Estifanos Shargie, Ryuichi Komatsu, Paul Smithson, Julie Thwing, Jules Mihigo, Samantha Herrera, Cameron Taylor, Jui Shah, Eric Mouzin, Steven S. Yoon, S. René Salgado

**Affiliations:** 1 President’s Malaria Initiative, U.S. Agency for International Development, Washington, District of Columbia;; 2 Malaria Branch and President’s Malaria Initiative, Centers for Disease Control and Prevention, Atlanta, Georgia;; 3 The DHS Program, ICF International, Rockville, Maryland;; 4 MEASURE Evaluation, ICF International, Rockville, Maryland;; 5 The Global Fund to Fight AIDS, Tuberculosis and Malaria, Geneva, Switzerland;; 6 Ifakara Health Institute, Dar es Salaam, Tanzania;; 7 President’s Malaria Initiative, Centers for Disease Control and Prevention, Bamako, Mali;; 8 Roll Back Malaria Partnership, Geneva, Switzerland

## Abstract

As funding for malaria control increased considerably over the past 10 years resulting in the expanded coverage of malaria control interventions, so did the need to measure the impact of these investments on malaria morbidity and mortality. Members of the Roll Back Malaria (RBM) Partnership undertook impact evaluations of malaria control programs at a time when there was little guidance in terms of the process for conducting an impact evaluation of a national-level malaria control program. The President’s Malaria Initiative (PMI), as a member of the RBM Partnership, has provided financial and technical support for impact evaluations in 13 countries to date. On the basis of these experiences, PMI and its partners have developed a streamlined process for conducting the evaluations with a set of lessons learned and recommendations. Chief among these are: to ensure country ownership and involvement in the evaluations; to engage stakeholders throughout the process; to coordinate evaluations among interested partners to avoid duplication of efforts; to tailor the evaluation to the particular country context; to develop a standard methodology for the evaluations and a streamlined process for completion within a reasonable time; and to develop tailored dissemination products on the evaluation for a broad range of stakeholders. These key lessons learned and resulting recommendations will guide future impact evaluations of malaria control programs and other health programs.

## INTRODUCTION

Over the past decade there has been an intensified effort in malaria control in malaria endemic countries from Ministries of Health (MoH) with support from international partners and funding agencies. Given the level of investment and effort in malaria control, members of the Roll Back Malaria (RBM) Partnership, including MoHs and their national malaria control programs (NMCPs), were interested in assessing the impact of their malaria control efforts. Information on the effectiveness of malaria control measures could impact future funding for malaria control and prevention and assist in prioritizing the use of those funds. This led the RBM Monitoring and Evaluation Reference Group (RBM MERG) to publish an initial methodology framework for conducting impact evaluations of malaria control programs in 2007,^1^ and to discuss key principles at an international multiagency workshop on impact evaluation in Tanzania in 2010. The President’s Malaria Initiative (PMI) has been working with host country governments, research institutions, and other national and international partners to conduct a series of impact evaluations in malaria endemic countries. To date, evaluations have been conducted in Angola, Malawi, Mali, Mozambique, Rwanda, Senegal, Tanzania (both for mainland Tanzania and Zanzibar) and Uganda, with evaluations underway in Democratic Republic of the Congo, Ethiopia, Kenya, and Liberia. The methodology framework for conducting an impact evaluation of national malaria control efforts is described in detail in the RBM MERG Impact Evaluation Framework^2^ and Yé and others in this supplement.^3^ This article describes the operational details for organizing and conducting the evaluations, the lessons learned, and recommendations for future impact evaluations.

## EXPERIENCE CONDUCTING MALARIA IMPACT EVALUATIONS

**Figure 1.**
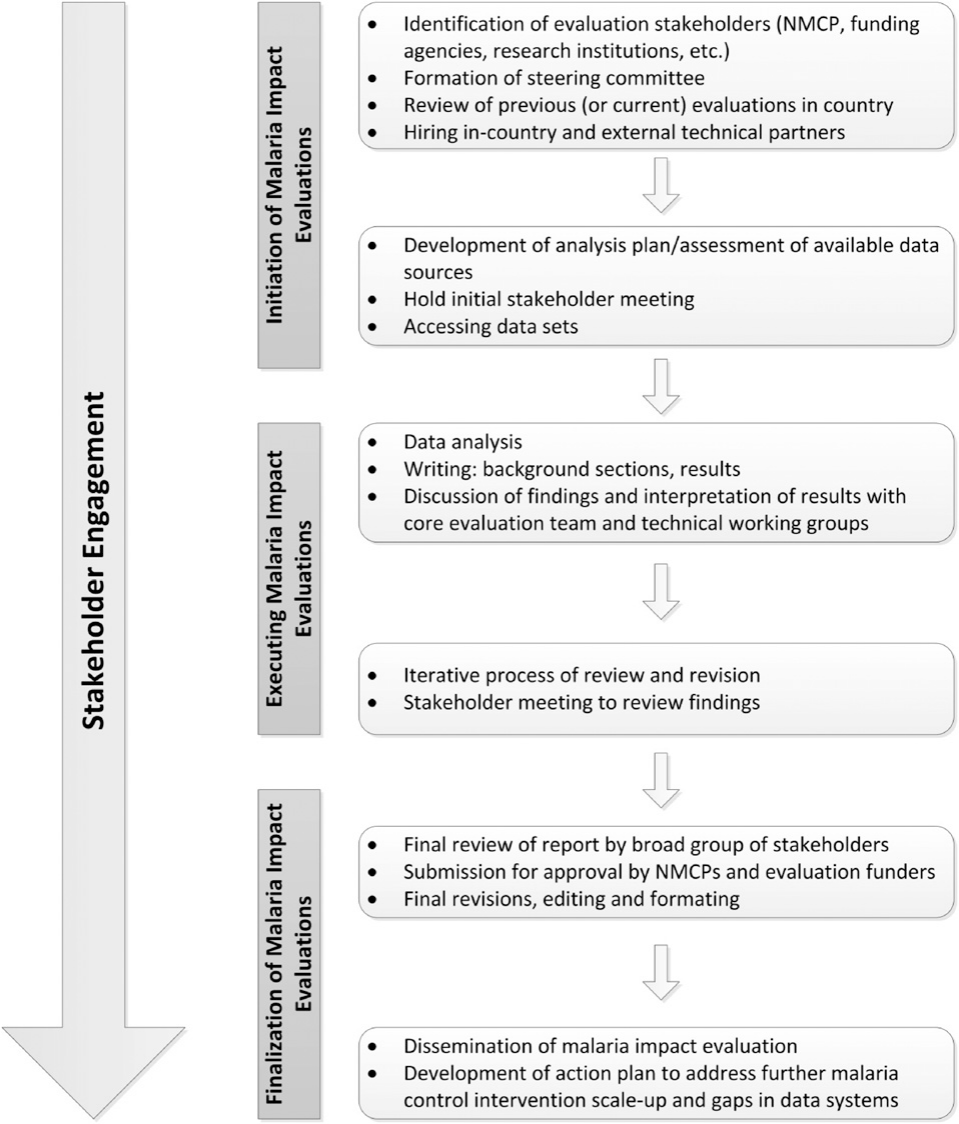
Framework for conducting malaria impact evaluations. Impact evaluations of the malaria control programs generally take 12 months to conduct and can be divided into three phases: initiation, execution, and finalization of the evaluation. Specific activities occurring in each phase are shown. Stakeholder engagement occurs throughout the evaluation, with specific groups of stakeholders brought in at various times.

The malaria impact evaluations conducted to date followed the same general process (Figure 1) with common, but in some cases unique, challenges (Table 1). The process included three phases: initiation, execution, and finalization of the evaluations. The initiation phase involved agreement between the NMCP and funding agencies to conduct an evaluation, identification of evaluation stakeholders, hiring of a local (and external) implementing partner, and holding an initial stakeholder meeting to introduce and get buy-in for the evaluation. The initiation phase also included identifying data sources, developing an analysis plan, obtaining institutional review board (IRB) approval/ethical clearance from relevant authorities, and accessing data. The second phase (execution) consisted of the data analysis, interpretation, and writing of the evaluation report. Stakeholders also reviewed and edited multiple drafts of the impact evaluation reports. During the finalization phase, the evaluation report was sent for review by all partners before finalizing and submitting for approval to the NMCP and PMI. In many cases, a stakeholder consultative meeting was held in country to review evaluation findings before submitting for approval. The process for conducting these evaluations took more than a year in all countries.

**Table 1:** Challenges and recommendations for conducting malaria impact evaluations

Issue/challenge	Recommendations
Overarching issues	
Need to generate buy-in and ensure transparency	• Involve all relevant stakeholders throughout the evaluation process
Need to involve many stakeholders but also keep the process streamlined	• Designate three groups of stakeholders with clear roles and responsibilities throughout the evaluation: steering committee, broad stakeholder group, core evaluation team
	• Engage the broad stakeholder group when needed (e.g., presentation of findings), but rely on smaller groups of stakeholders (e.g., technical working groups) to focus on a particular technical area
Evaluations often take more than a year, making it difficult to maintain stakeholder engagement	• Make every effort to conduct the evaluation within a one-year time frame
	• Generate buy-in and reach an agreement on the importance of the evaluation at the outset
Potential conflict of interest when the evaluations are led by the NMCP and/or partners	• Consider hiring an independent evaluation team
	• Agreement to release the findings whether there is demonstrated impact or not
Initiation phase	
Determining when it is appropriate to conduct an impact evaluation	• Discuss with the NMCP, funding agencies and steering committee before commencing the evaluation
	• Consider epidemiological context, available quality data, timing and extent of malaria control intervention scale-up (see decision tree, Figure 2)
	• Consider postponing the evaluation or conducting a program review or coverage assessment if the conditions are not met for a national-level impact evaluation
Identification of relevant data sources	• Comprehensive mapping and assessment of existing data, and access to data sources should be discussed prior to and during initial broad stakeholder meetings
Multiple partners or funding agencies interested in conducting an evaluation during the same time period can burden the country or cause confusion	• Where possible, partners should collaborate to conduct one joint evaluation
	• Where more than one evaluation makes sense, partners should share findings and coordinate the dissemination of the evaluations
Need for financial and human resources for conducting the evaluation	• At the outset partners should agree to their level of funding or in-kind contributions to the evaluation
	• Include funding for any capacity strengthening activities and the final dissemination (e.g., event, printing of full reports or key findings reports)
Execution phase	
Identifying the right combination of partners and particularly the lead partner for the evaluation	• Identify a lead for the evaluation with the NMCP (and steering committee)
	• Consider the capacity and time availability of the NMCP, local and external technical partners when selecting a lead
	• Bring in additional technical assistance as needed
	• Designate a dedicated core evaluation team to conduct the evaluation and focus on the day to day activities
Finalization phase	
Stakeholder disagreement over results or conclusions	• Provide ample opportunity for review and discussion
	• If there are contradictory findings conclude that results are inconclusive with regards to a particular aspect of the evaluation
	• The stronger the methods are and the less room for interpretation should reduce disagreement
Evaluation clearance was time consuming	• Establish clear procedures for clearance of the evaluation reports and associated documents at the outset of the evaluation
Generating evaluation products that will be of use to multiple stakeholders	• Generate multiple products from the evaluation including a full core report with annexes, key findings report, journal article(s), policy brief, etc.
Need to make the impact evaluation results useful to the NMCP and partners	• As part of the dissemination activities an action plan should be developed by all partners to address intervention coverage gaps
	• Address data gaps in the action plan, which will improve the evaluation process in the future, including planning for a prospective evaluation
The final documents need to be available in both the official local language (e.g., French or Portuguese) and English	• Plan time and funding for translation services
	• Provide French (or Portuguese) versions of analysis plans, protocols, and report outlines
	• Consider producing the key findings report in the official language and English, but the full core report only in the official language

NMCP = national malaria control program.

Based on the experiences with these initial thirteen countries, the evaluation teams generated positive lessons learned and have identified various challenges and bottlenecks to the process that, if addressed, can lead to timely completion of impact evaluations and generation of high quality reports.

### Stakeholder engagement.

Stakeholder engagement is the first step toward production of a robust, collaborative, locally relevant evaluation in which partners engage in the process and make use of the evaluation findings.^4^ It is important to ensure there is a common understanding of the purpose and objectives of the evaluation, including the process and the methods that will be used to describe malaria intervention expansion over time methodically and to demonstrate how these improvements may have contributed to declines in malaria morbidity and all-cause mortality over the same period. Engaging stakeholders early and often (Figure 1) provides transparency, generates buy-in, enables information sharing, and helps to ensure the evaluation responds to stakeholders’ needs. Stakeholder involvement is also important for identification and access to data, interpretation, validation, and dissemination of results.

#### Experience and lessons learned.

A broad range of stakeholders were invited to participate in the evaluations, but it was critical to have the MoH/NMCP involved, and where possible to lead the evaluations. Malaria impact evaluations were included in some NMCP’s Monitoring and Evaluation (NMCP’s M and E) plans. From the experience in conducting the evaluations to date, it was crucial for the MoH/NMCP to appoint a representative from the NMCP to serve as a point of contact for the evaluation, especially when the NMCP was not leading the evaluation. For example, in Malawi and Liberia the NMCP’s M and E focal points were involved in all discussions and decisions related to the evaluations. Including stakeholders in addition to the NMCP and funding agencies was particularly valuable when stakeholders were aware of studies done in country and could provide access to the reports and the data or could provide information on contextual factors (e.g., other health interventions) during the evaluation period.

Experience from the initial evaluations showed that stakeholders could be divided into roughly three groups based on when they were involved and their level of engagement in the evaluations: steering committee, broad stakeholders group, and core evaluation team (Figure 1 and Table 1). First, was the steering committee, although this was not always formalized. A steering committee was responsible for advising on directions for the evaluations in Senegal and Mali and also participated in the development of the request for proposals for the consultants for the Senegal evaluation. In countries without a formal steering committee, the NMCP and partners also filled these roles and were responsible for submitting an IRB proposal. In countries where there was a steering committee, it approved the evaluation protocol before submitting to the IRB. The steering committee was also consulted toward the end of the evaluation for review of the final evaluation report. Second, was the broad group of stakeholders who met at the outset of most of the evaluations to help brainstorm ideas around the evaluation, including identifying published and unpublished research studies, case studies, and also identifying pertinent data. It was important to engage the broad group of stakeholders early in the initiation phase. This was conducted at slightly different times in Liberia and Mozambique compared with Rwanda, but in all cases served to initiate the process and generate buy-in. In Liberia and Mozambique, stakeholders met at the outset of the process before any data analysis began and the meeting was used to generate ideas on other data sources (besides household surveys) to include in the analysis. The initial stakeholder meeting was conducted in Rwanda after the survey data had been analyzed. A workshop was held with the Rwandan NMCP, funding agencies, and external and local technical partners to review the preliminary findings, to identify additional data needs, and to discuss coordination of the report writing efforts. In most countries, the broad stakeholders group met again toward the end of the evaluations to review the results and assist with interpreting the findings, which fed into the final report.

In addition to the broad stakeholders group, a very successful approach was to form technical working groups with subsets of the stakeholders who advised on specific aspects of the evaluation. In mainland Tanzania, the local implementing partner convened a number of small technical working groups to review evidence available on topics including malaria in pregnancy, case management, vector control, and facility-based health data. Similarly in Zanzibar, expert working groups were convened to provide topic-specific guidance for the evaluation. Other countries did not constitute these technical working groups but instead reached out to individual experts in the field to provide the necessary data or contextual perspective.

The final group of stakeholders was the core evaluation team, which was involved in the day-to-day conduct of the evaluation. The composition of the core evaluation team varied by country, but included the NMCP point of contact, and representatives of funding agencies, local and external technical implementing partners, and in some countries, additional in-country partners.

In all countries, one major challenge was keeping the stakeholders engaged. This was addressed by having regularly scheduled phone calls and follow-up e-mail communications during the evaluation to discuss progress, process, and methodological issues, and to review results. The core evaluation team was involved in these routine activities and all stakeholders were continuously provided updates through e-mail communications. However, despite these efforts, it was difficult to retain the same level of engagement of the broad group of stakeholders, and in some countries of the core evaluation team, throughout the evaluation process.

#### Recommendations.

- Establish a steering committee for the evaluation to guide the evaluation process, approve the evaluation protocol and assist in identification of a local implementing partner. If no formal steering committee is formed, the NMCP and key in-country partners will need to initiate the evaluation and if needed submit the evaluation protocol for IRB approval. The evaluation team should use any existing in-country arrangements to assist with these activities.
- The NMCP should lead the evaluation if possible, but where this is not possible the NMCP should appoint at least one staff member to serve as a point of contact for the evaluation who will work as part of the core evaluation team. Malaria impact evaluations should be included in NMCPs’ M and E plans.
- Engage a broad group of stakeholders at the initiation, results review and dissemination stages of the evaluation. Use (or establish if necessary) technical working groups to provide advice on specific topics during the evaluation.
- The core evaluation team should periodically update the MoH/NMCP, funding agencies, and other key stakeholders.
- Engage focused technical stakeholders and partners who can provide information and context for the implementation of the malaria control efforts in country and are familiar with nonmalaria health programs in country.

### Partner coordination.

Given the increasing demand from governments, partners and major funding agencies for impact data for malaria program evaluation and resource allocation decisions, the need for coordinated evaluation efforts is paramount. It is important to address the needs of the NMCP as well as partners. In conducting malaria impact evaluations, the goal should be to coordinate among the various partners to avoid multiple evaluations, where possible. The duplication of efforts poses an unnecessary burden on the time and resources of the NMCP and other stakeholders. In addition, collaboration on one, joint evaluation effort when possible provides a forum for consensus on methods, approaches, and results.

#### Experience and lessons learned.

It was valuable having multiple partners work together on the impact evaluations; and where it was possible to coordinate among the partners, there was a reduction in the number of similar evaluations in country. In Ethiopia, PMI and the Global Fund to Fight AIDS, Tuberculosis and Malaria (Global Fund), in agreement with the government of Ethiopia, conducted a joint evaluation in 2013. The coordination was established by including members of both agencies in the planning, implementation, and review of the evaluation. PMI funded and organized the evaluation in Ethiopia looking at impact through 2013 and preparations are underway now for Global Fund to fund the next impact evaluation in Ethiopia in 2017. The Global Fund Technical Evaluation Reference Group emphasizes the importance of partner approaches to evaluation, recommends and supports joint evaluations in countries, and synthesizes evidence from those joint evaluations for global level strategic reviews.^5^ These country evaluations can be, or can complement, national program reviews with a robust assessment of epidemiology and program gaps and form the basis for preparation of funding concept notes to the Global Fund, and reports on progress of grant implementation.^5^

There were some instances where multiple evaluations were unavoidable due to different requirements or methodologies. For example, in Malawi and Uganda, PMI funded impact evaluations^6,7^ and the UK’s Department for International Development funded epidemiological profile reports^8,9^ at the same time. These reports differed in approach and content, yet there were complementary elements. Some of the data from the epidemiological profile reports were used as an input to the PMI-funded impact evaluations, but there was no overall coordination. Learning from the experiences in Malawi and Uganda, when the evaluation was designed in Kenya, efforts were made to coordinate the two evaluations by including some members on both evaluation teams and building off each other’s evaluations. Another example of concurrent evaluations was in Uganda where PMI and the Institute of Health Metrics and Evaluation (IHME)^10^ (funded by the Bill and Melinda Gates Foundation) conducted evaluations. The PMI-funded evaluation followed the plausibility argument approach proposed by the RBM MERG and IHME selected an approach to address causal inference. The two evaluation groups were in communication and reviewed and referenced the others’ reports.

In several countries, the RBM Executive Director sent letters to the MoH/NMCPs to introduce the impact evaluations and request their participation. This served to indicate to the countries that the broader RBM Partnership was supportive of these evaluations and it was not the effort of only one funding agency. Requirements of the NMCPs were included in the planning of the evaluations, including agreements on publications resulting from the evaluations and sections of the report the NMCP wanted to write. The methodology was also discussed and agreed upon with the NMCPs.

#### Recommendations.

- Partners should support countries in their efforts to evaluate the impact of their malaria control programs and should collaborate to conduct one coordinated evaluation, whenever possible. NMCP requirements also need to be included.
- Partners should discuss planned evaluations in an attempt to coordinate the evaluations or conduct a single joint evaluation. Ideally, there would be a forum where partners could present plans for these evaluations and several venues are possible. For example, globally through the RBM MERG or through donor meetings or malaria coordinating committees in country.
- Where multiple evaluations are deemed necessary, efforts should be made to coordinate review of the results and dissemination of findings to avoid confusion and conflicting messages in country.

### Designing an evaluation.

The PMI-funded evaluations were conducted using an ecological plausibility evaluation design as recommended by the RBM MERG.^1–3^ There are several aspects to consider when designing an evaluation, including availability and quality of data, epidemiological context (transmission pattern and intensity, phase of control), timeline and magnitude of program scale-up, and recent evaluations or assessments, if any.

#### Experience and lessons learned.

A common method^1–3^ can be applied to evaluate the impact of malaria control interventions in high burden countries with adaptations for the specific country context. Within a given evaluation framework, it was important to consider the availability of high-quality data sources, epidemiological context, and level and duration of intervention implementation when choosing an appropriate design. Alternative evaluation approaches have been proposed.^2,3^ The standard methodology consisted of building a plausibility argument through assessing trends of malaria intervention coverage, trends of malaria morbidity and under-five mortality, and trends of key contextual factors potentially affecting child survival. However for countries where data allowed, additional advanced analyses including cox-proportional hazard regression were done to quantify the effect of malaria interventions (e.g., insecticide-treated net ownership) on child survival.^2,3,11^

Information about the malaria control program was gleaned from the National Malaria Strategic Plans, the country’s Annual Statistical Reports, Malaria Program Reviews (MPRs) and partner reports (UNICEF, PMI, etc.). MPRs provided information on the program activities, implementation progress against national strategic plan targets, and the potential for impact.^12^ MPR reports were used in conjunction with analytic results from impact evaluations to provide additional contextual information on how well the program was implemented, which assisted in interpreting the evaluation results. The impact evaluations can be complementary to the MPRs.

**Figure 2.**
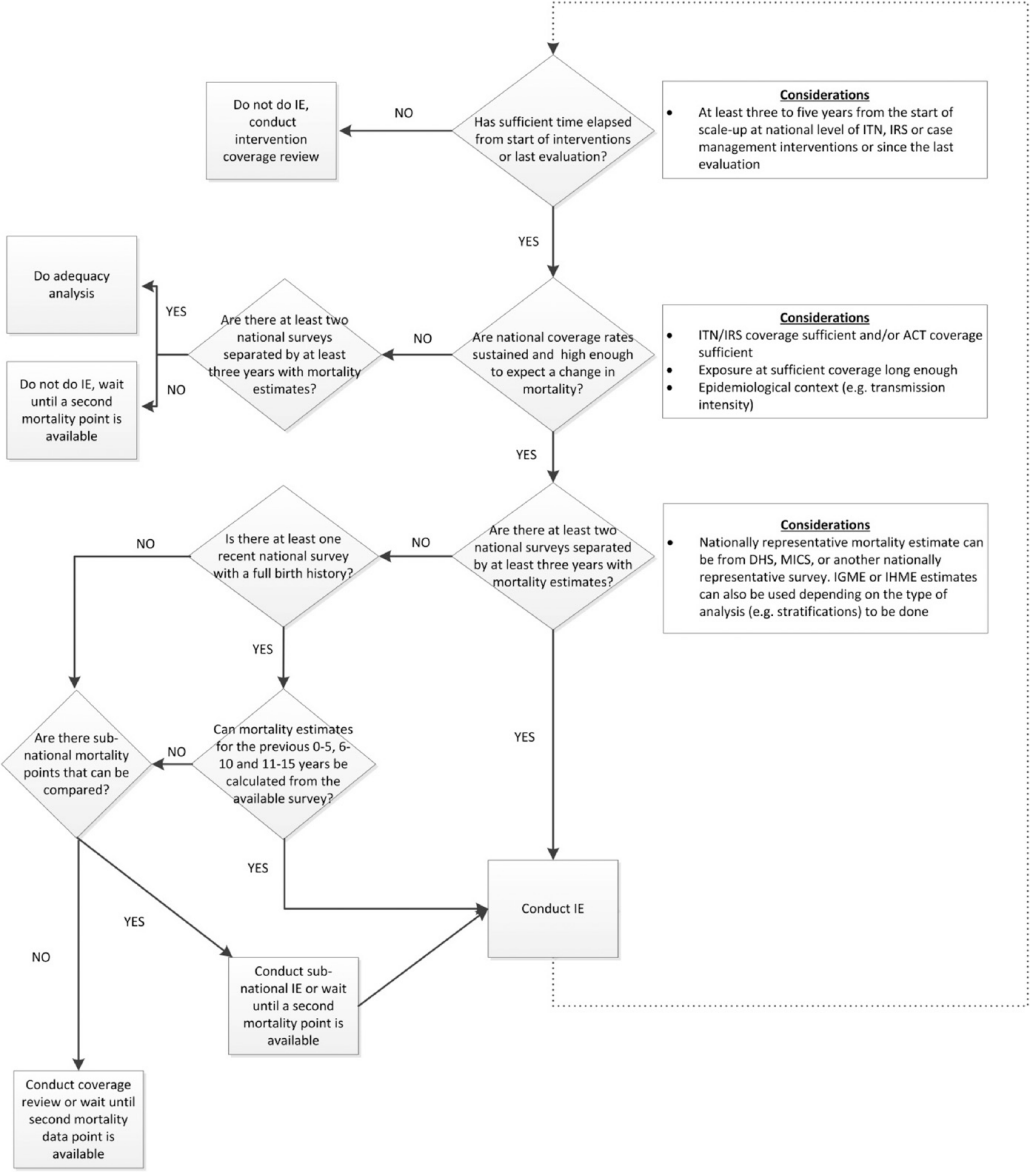
Malaria impact evaluation decision process. When deciding when it is appropriate to conduct a national-level impact evaluation several considerations need to be taken into account including epidemiological context, intervention coverage levels, time since intervention scale-up began, time intervention coverage levels have been sufficiently high, and time since last evaluation. Several alternatives to a full national-level impact evaluation are provided. This decision process is applicable for high burden countries using all cause childhood mortality as the primary impact measure. DHS, Demographic and Health Survey; IE, impact evaluation; IGME, UN Inter-agency Group for Child Mortality Estimation; IHME, Institute for Health Metrics and Evaluation; MICS, Multiple Indicator Cluster Survey.

Using lessons learned from the initial evaluations in Tanzania, Malawi, and Angola, a process for determining if it was appropriate to conduct a national-level impact evaluation with all-cause childhood mortality (ACCM) as the key impact indicator was developed (Figure 2). It should be noted there are no definite thresholds for what level of malaria control intervention coverage is necessary to expect national-level impact, nor for how long this coverage needs to be sustained until impact could be expected on malaria morbidity and mortality, although several studies have modeled this.^2,3,13^ However, by using ACCM as the key impact indicator (5-year estimate), interventions at least need to be implemented and coverage maintained in advance of the midpoint of this 5-year period, which has proven to be a challenge in these initial evaluations. An increase in intervention coverage only in the last year of the 5-year period covered by the ACCM estimate is likely to have an impact that year, but this may not translate into impact seen within a 5-year mortality estimate. It is also important to note that impact could still be achieved subnationally if intervention coverage is uneven, but the analyses will likely need to be stratified by geographic location to address this. On the other hand, if the intervention coverage is uneven, but targeted to the highest burden areas, this may impact national mortality estimates.

The importance of evaluation timing was evident in the case of the Angola evaluation. In 2002, Angola emerged from a civil war in which much of its infrastructure, including health infrastructure, was destroyed. The NMCP in Angola had been scaling up its malaria control interventions since emerging from the war, but the evaluation revealed that intervention coverage was not at a level where national-level impact on ACCM could be expected.^14^ In response to the determination that the evaluation was premature, an attempt was made to focus on one of the provinces where many malaria control activities were focused. In retrospect, an evaluation of progress in intervention coverage may have been preferable to the full impact evaluation assessing impact on ACCM that was conducted. Technical challenges of a different nature were experienced in Ethiopia,^15^ where using ACCM as the primary impact measure may not have been the best approach given the heterogeneity in malaria burden, with some areas experiencing a high level of malaria transmission and other areas experiencing very little. A national evaluation using ACCM masked a lot of this difference as reliable estimates of ACCM restricted to just those areas with malaria risk were not available.

The experience from conducting the evaluation in mainland Tanzania emphasized the need for an agreed-upon analysis plan at the outset of the evaluation. This evaluation was the first one completed following the plausibility evaluation approach.^16,17^ The final set of analyses, including indicator stratifications,^2,3^ were determined through an iterative process throughout the evaluation implementation. General guidance documents generated at the outset of the process in Tanzania were updated with lessons learned from that evaluation and then used to inform subsequent evaluations. The core evaluation team sought advice from external experts in the fields of malaria and child health program evaluation through a technical advisory group. This group reviewed the mainland Tanzania impact evaluation report and guidance documents, and met with evaluation funders and implementers at a 3-day meeting to revise the methods for the subsequent evaluations. On the basis of these recommendations, a more detailed standardized evaluation design with a detailed analytical plan was developed, which was first implemented in Uganda and then further adapted for the subsequent evaluations.

A limitation of the malaria impact evaluations conducted to date has been that they were all retrospective. The evaluation team therefore used what data was available, instead of being able to plan data collection to best measure impact. As discussed below under the use of the evaluation results, it is recognized that one of the results of the evaluation is the identification of gaps in the available data. NMCPs and partners have been encouraged to strengthen their data systems as a result.

#### Recommendations.

- Review the availability and quality of data, malaria epidemiology, and timing and level of malaria control intervention coverage (e.g., what level of coverage has been reached and how long has coverage been at this level) prior to the design phase of an evaluation^2,3^ to determine if a national-level impact evaluation is appropriate.
- A standard methodology^2,3^ and operational framework was developed and can serve as a guide for future impact evaluations. It is recommended that evaluators implement this approach, reviewing and adapting for the country context as needed. Where data allow, additional advanced analyses can be conducted to quantify the effect of malaria control interventions on child survival.
- Although an analysis plan needs to be agreed upon at the outset to guide the evaluation, it is equally important to also have some degree of flexibility to adjust the analytical plan based on any new evidence that emerges during the course of the evaluation, including interim findings from the ongoing evaluation.
- The evaluation approach was designed for estimating impact in high malaria burden countries, using ACCM as the primary impact indicator. There is a need to develop an impact evaluation approach suitable to moderate and low transmission settings and discussions on such a methodology are underway.
- Prospective evaluations are ideal and provide the evaluators the opportunity to collect data prospectively thus overcoming the limitations of relying on data already available. Particularly for countries looking at conducting another malaria impact evaluation in the future, plans for improving (or adding) data sources should be developed following the completion of the first evaluation.

### Data access.

The strength and depth of impact evaluations depends on the type of data available for the evaluation. Accessing the appropriate data sources with sufficient quality for an impact evaluation was one of the more time consuming steps. Identifying the data sources, gaining access to the data, and reviewing the data quality took a substantial amount of time. However, this was an extremely important aspect in ensuring all relevant data were identified and thoroughly analyzed to draw meaningful conclusions.

#### Experience and lessons learned.

In addition to large household survey data, other data sources added depth and completeness to the evaluations. Sources included data from anemia and parasitemia surveys, Health and Demographic Surveillance Systems (HDSS), and Health Management Information Systems (HMIS), including sentinel surveillance sites. In addition, information on socioeconomic conditions, nonmalaria health interventions and meteorological (rainfall and temperature) data were considered.^2,3^ When requesting access to data it was important to reach an understanding with the party that holds the data, regarding who will have access to the raw data and its role in the evaluation. For most of the evaluations, complete data sets were provided, but in others the owner of the data performed the requested analyses for the evaluation team without sharing the data. For example, in Malawi, researchers at the National Center for Chronic Disease Prevention and Health Promotion at the Centers for Disease Control and Prevention provided and reanalyzed parasitemia results from the National Micronutrient Surveys.^6^ In some instances, a memorandum of understanding (MOU) was requested before access to data was granted. For example, a written request was submitted to the NMCP in Malawi for access to the 2010 MIS dataset. Ideally, MOUs regarding data access (specifically in reference to making the data publically available) should be developed at the time a survey or study is conducted.

Another solution to the data access issue was having an impact evaluation analyst work with the holders of the data. This approach was used in Zanzibar, where one of the consultants working on the evaluation went to Zanzibar and analyzed the HMIS data in conjunction with the Zanzibar Malaria Elimination Program. This had the added benefit of strengthening capacity for data analysis and evaluations within the malaria control program.

In some countries, the evaluation team was unable to gain access to the data. It was important to move forward with the evaluation using the main data sets, including household surveys and HMIS where possible, and not hold up the evaluation for the additional data. Where possible the evaluation referenced published reports when the data could not be analyzed directly.

#### Recommendations.

The evaluation team should:

- Begin working with the MoH/NMCP and the other stakeholders as early as possible to identify useful data sets, including malaria, other maternal and child health information, and meteorological data, and to request access to them.
- Establish a robust and transparent decision-making process for determining the quality of various data sets, with a clear decision point regarding whether to use the data in the evaluation. For national household survey data, review the methodology and pay particular attention to the sample size to make sure it is powered to measure key indicators in a sub-population or at sub-national level. Specific attention should be given to data from routine health information systems by setting a minimum cutoff point for data completeness. Regardless of the indicators or data elements, the level of completeness should be at least 85% to avoid random results which are difficult to explain over time.
- Identify alternative approaches when the raw data sets are not available or not of sufficient quality. Options include: request that select indicators be abstracted; request that the owners of the data sets perform the requested analysis for the impact evaluation; have a member of the evaluation team work with the NMCP or local organization to analyze the data in country; or provide data cleaning and analysis assistance to the owners of the data.

### Executing the evaluations—data analysis and writing.

The second phase of the impact evaluation process is the implementation/execution phase, which includes the data analysis, interpretation of results, and writing. In the course of conducting the initial impact evaluations, several different approaches were used based on local capacity and the NMCP’s availability as outlined below.

#### Experience and lessons learned.

A common evaluation methodology framework^2,3^ was developed and adapted to each country’s context. Similarly, a common operational framework was developed as described in this paper and adapted to each country’s capacity. In all evaluations, technical expertise in analyzing the large household survey data sets was provided by an external technical partner, but the evaluation lead and partner responsible for writing the reports varied by country. In some countries the NMCP led the evaluation; however, in others the NMCP remained engaged but preferred that the process be led by another partner.

In each country in which PMI initiated the process, PMI negotiated with the NMCP (and in some countries with the steering committee) to determine the best fit to accomplish the evaluation in a reasonable period of time, while preserving transparency and affording opportunities for all partners to contribute.

Although it was critical to complete the evaluation in a timely manner it was also important to take advantage of opportunities to build and strengthen local capacity. Capacity building of the NMCP and/or local institutions needed to be agreed upon at the outset, with the understanding that some of the analyses would be completed externally. Providing capacity strengthening opportunities through working closely with external technical assistance partners incurred additional time and costs and these needed to be planned for at the outset. For example, in Liberia the international technical partner organized a multiple day workshop with the NMCP and local technical partner to explain the impact evaluation methodology and to work through interpreting the analyses of the Liberia data.

Conducting the evaluations as a team resulted in an increased exchange of ideas, improved transparency, and capacity strengthening of local institutions and NMCP staff, but also contributed to lengthening the evaluation timeline. Although not explicitly defined at the beginning of these evaluations, there were three general implementation approaches that emerged:

##### NMCP-led process.

In Mali, Senegal and Zanzibar the NMCP led the evaluation process and brought in local and external technical partners to assist as needed. The NMCP in Senegal led the process and a local consulting firm was hired to help conduct the evaluation. The consultants worked directly with designated staff members from the NMCP and had an office within the NMCP for the duration of the evaluation to facilitate interaction. The NMCP, consultants, and the PMI/Senegal team working together were responsible for analysis, interpretation, and writing of the report with support and guidance from an international technical partner. In Mali, the local partner worked closely with the NMCP and the PMI/Mali team to organize technical meetings with key malaria partners, analyze the majority of the data, and write the report.

##### In-country technical partner as the lead.

In-country technical partners led the evaluation in a subset of countries. The local partner organized stakeholder meetings, conducted some of the analyses, and wrote the impact evaluation report; whereas, the external technical partner conducted the analysis of the household survey data. For example, in mainland Tanzania the evaluation was led by a local partner whose strong working relationship with the NMCP and other partners in malaria control in country facilitated collaboration across various expert groups and data sources. Similarly, in Kenya, the evaluation was led by an in-country partner with strong participation of the NMCP and a local research institution and with technical support from an international technical partner.

##### External technical partner as the lead.

In some countries (Angola, Liberia, Malawi, Mozambique, and Uganda), a local partner was hired to complete parts of the evaluation along with the NMCP, but the lead for the evaluation was an external technical partner. In each case, the local partner was asked to write the background chapters on the history of malaria control in the country and the roll out of the malaria control interventions. The local partners were critical for gaining access to data sources, analyzing some of the data, convening stakeholder meetings, and coordinating with the NMCP. For example, in Mozambique, the local partner worked closely with the NMCP to organize and implement two stakeholder meetings held at the beginning and end of the evaluation; wrote the background sections of the report; conducted the analyses for and wrote the further analyses section; and coordinated the review of the report drafts with local stakeholders. This local partner had a strong working relationship with the NMCP and was well positioned to manage the process locally. A local partner played a similar role in the evaluation in Liberia.

#### Recommendations.

- Identify national and international partners to complete a high quality evaluation in a reasonable period of time, based on in-country capacity and time available for the NMCP to work on the evaluation.
- Work with the NMCP (and steering committee) to identify the most appropriate partner to lead the evaluation, whether this is the NMCP, an in-country technical partner, or an external technical partner.
- Provide opportunities to improve technical capacities of the NMCP and/or local implementing partner. Budget time and funding for agreed upon capacity strengthening activities at the outset of the evaluation.
- Ensure in-country technical partners have strong ties with the MoH/NMCP and are able to work with the external technical partner.

### Dissemination and use of evaluation findings.

The malaria impact evaluations provide information on whether there has been an overall decline in ACCM (and in some cases malaria-specific mortality) and whether these declines could plausibly be due in part to the expansion of malaria control intervention coverage. The impact evaluations are not designed to provide information on the success of individual malaria control projects or interventions, nor of the leadership or management functions of the malaria control programs. If there is demonstrated impact, it provides the MoH/NMCPs, partners, and funding agencies with justification for continued support for scale-up and/or maintenance of malaria control interventions. Likewise, if there is no measured impact or if the analysis is inconclusive, this should trigger further investigation into the reasons why no impact was identified and a re-evaluation of both the appropriateness of the interventions and the implementation approaches.

#### Experience and lessons learned

#### Dissemination of the findings.

The presentation of impact evaluation results needs to be tailored to the audience. In most countries, there were multiple audiences that benefited from and used the results, necessitating the production of multiple documents. The evaluation teams found that lengthy, detailed impact evaluation reports were not ideal for advocacy nor for the malaria community. As a result, after the first set of evaluations the impact evaluation reports were streamlined, while still retaining the detailed methods and analyses in a set of annexes to the report. In addition to the impact evaluation reports themselves, in some countries the results from the impact evaluations were used to produce two additional types of documents: RBM Progress and Impact (P and I) Series Country Focus reports^18,19^ and scientific journal articles.^11,16,20–22^ The RBM P and I reports, which were advocacy-focused documents, afforded an opportunity to present findings from the impact evaluation in a clear and concise format, to tailor the report to a wider audience (e.g., politicians, media, advocates, development partners), and to share lessons learned during the scale-up phase of malaria control interventions in a given country. Results from the evaluation in mainland Tanzania were also used as a country showcase for impact in the Global Fund results reports.^23^

Dissemination events (e.g., high-level events where the report was released) were held in both mainland Tanzania and Malawi surrounding the release of the RBM P and I reports^18,19^ and in Senegal around the release of the core report.

For dissemination of the results in country (when the dissemination was not part of an RBM P and I report release), the evaluation team developed a summary of the key findings. This took the form of an expanded executive summary and was used for the dissemination events in Senegal, Uganda, and Kenya. This format presented the results of the full impact evaluation in a more digestible way for a broad range of stakeholders.

#### Use of the findings.

National malaria control programs used the results to provide evidence of the impact of their malaria control program. Funding agencies (e.g., PMI and Global Fund) used the findings as part of their reporting requirements to demonstrate the contribution of their support to the impact of malaria control and also to justify sustained funding of the malaria programs. The results of the impact evaluations were used by countries to report against the Millennium Development Goals and, more recently, the Sustainable Development Goals (SDGs). Donor agencies and the malaria community have used the results of the impact evaluations to assess progress toward national and global malaria control targets. The impact evaluation results could also provide epidemiological data to feed into the MPRs.

National malaria control programs and partners also used these evaluations to identify gaps in their data collection systems. In some countries, it was not possible to analyze the routine data or the multiple routine data sources gave conflicting trends. For example, in one country aberrant malaria mortality findings from the routine data resulted in a team from the NMCP and MoH visiting health facilities to review the data to understand how malaria deaths were being captured in the routine information system. As part of the dissemination process, malaria control programs and their partners were encouraged to review the results and limitations of the evaluation and develop an action plan for building off the current malaria control efforts in country, including identifying gaps in malaria control interventions and gaps in data systems.

#### Recommendations.

- As appropriate, use multiple formats to present the impact evaluation findings (e.g., full report, key findings report, journal article, and/or policy brief) to meet the needs of the various stakeholders. A detailed scientific report can be produced and serve as the main reference report for the other documents released in association with the evaluation. Consider a key findings report for World Malaria Day events or to accompany the dissemination of the final report.
- Prepare journal articles and/or present at scientific meetings to inform the broader malaria community. Peer review and publication of the evaluation findings provides further validation of the impact evaluation results and creates buy-in.
- Impact evaluations can be used to report progress against targets of the SDGs and as a key input to MPRs and Global Fund applications.
- The evaluation process will likely uncover gaps and limitations in the data. These should be noted and discussed with partners during the evaluation, and at the end of the evaluation an action plan should be developed as part of the dissemination process to strengthen the data, especially routine data collection systems in country. This can inform prospective planning of future evaluations to better meet the needs of NMCPs and partners.
- The results of the evaluation should also be used by NMCPs and partners to strengthen their program in response to low levels of intervention coverage. If there is no demonstrated impact the NMCP and partners should investigate the root causes of this and address these to strengthen their programs.

## ADDITIONAL ASPECTS OF CONDUCTING MALARIA IMPACT EVALUATIONS

Outlined below are additional considerations when planning a malaria impact evaluation. Although not unique to impact evaluations, they are important to successfully completing these evaluations.

### Evaluation process timeline.

Evaluations conducted following the RBM-MERG framework have taken a year or longer to complete. The longer timelines have been due to a lengthening of one or more of the three phases.

#### Experience and lessons learned.

In the initiation phase, additional time was needed in some countries primarily due to the identification and hiring (procurement process) of a local technical partner. The execution phase was prolonged because of the time consumed by multiple partners in reviewing numerous drafts of the core report, competing priorities for the NMCP and partners, and inaccessibility of data. Finally, delays have occurred during the final stage of review and clearance of the evaluation report.

The experience with the first thirteen evaluations has shown that any longer than a year and the evaluation became less efficient and risked being less useful to stakeholders. As discussed above, it is difficult to maintain stakeholder engagement over a long period of time. PMI with its partners have undertaken efforts to streamline the process for the subsequent evaluations to keep them near a 12-month time frame (Table 2).

**Table 2:** Suggested timeline for conducting malaria impact evaluations

Activity	Estimated time (week)	Partners involved
Initiation phase		
Start discussions with NMCP, in-country and international stakeholders	4	Funding agencies, PMI in-country teams, national authorities, steering committee
Contract local and external technical partners and identify any remaining members of core evaluation team and steering committee, agree on TOR (including facilitating data access)	6	Funding agencies, core evaluation team, steering committee
Develop work plan, analysis plan, report outline, and task matrix	2	Core evaluation team
Kick off the evaluation with a stakeholder meeting	1	Core evaluation team, funding agencies, national authorities, steering committee, broad stakeholder group
Gain access to data sets	2	Core evaluation team, broad stakeholder group
Execution phase		
Conduct preliminary analyses	4	Core evaluation team, discussions with technical working groups
Complete remaining analyses	4	Core evaluation team
Develop complete draft report	6	Core evaluation team
Evaluation team and identified stakeholders to review draft report	3	Core evaluation team, identified stakeholders
Convene consultative meeting to present the preliminary results	1	Broad stakeholder group, evaluation team, steering committee
Develop final draft report, incorporating feedback	4	Core evaluation team
Finalization phase		
Broad review of the final draft, allow external reviewers to comment on report	4	Broad group of stakeholders and any additional external reviewers
Complete final edits, proofreading, and formatting	3	Evaluation team, editor, proofreader, graphic designer
Final approval (in-country and funding agency clearance) and make necessary revisions	8	Funding agencies, national authorities, core evaluation team
Hold dissemination event to share findings	1	Evaluation team, funding agencies, national authorities, all stakeholders

NMCP = national malaria control program; PMI = President’s Malaria Initiative; TOR = terms of reference.

Modified from Roll Back Malaria Monitoring and Evaluation Reference Group impact evaluation framework^2^.

#### Recommendations.

- Concrete steps (Table 2) need to be taken to ensure the evaluations are completed within a year.
- It is recommended that a clear management/decision-making/communication strategy among partners be established at the outset. In addition, clear procedures for clearance and approval of the reports and associated documents need to be established up front.
- Limit the number of drafts of the report that key stakeholders (core evaluation team and additional identified stakeholders) are asked to review to the first complete draft and the final draft, with the core evaluation team reviewing specific sections in the interim.

### Language requirements.

Evaluations are of more use and more transparent when conducted in the official local language, which can bring with it a set of challenges.

#### Experience and lessons learned.

The evaluation in Senegal was conducted in French with the analysis plan, protocol, and all drafts of the report produced in French. Additional time was needed at the outset to translate the standard analysis plan and protocol to French. When it came time to finalize the report, there was a requirement from some partners to review the report in English. This required a significant amount of time to translate the technical document from French to English and then resulted in the need to make simultaneous edits to the French and English versions of the report. A similar situation arose in Angola, where the initial drafts of the background chapters were written in Portuguese. Given the need to review the report throughout the process in Angola, the core evaluation team switched all drafts to English part way through the evaluation.

#### Recommendations.

- Decide at the outset the language(s) the final report and associated documents will be produced in. One solution is to produce the key findings report in the official language and English, but maintain the full report in the official language.
- Translation services need to be budgeted for both in terms of the time needed for translation of documents and also the cost.

### Resource needs.

Conducting the malaria impact evaluations has required financial and human resources, which need to be addressed, planned, and budgeted for at the outset of an evaluation. The available human and financial resources should shape the type of evaluation that can be undertaken.

#### Experience and lessons learned.

#### Funding.

A detailed budget that includes costs for human resources, stakeholder meetings, payment for data sets, subcontracts, translation, and dissemination was prepared before the evaluations were implemented. The cost for an evaluation using in-country and external technical partners ranged from $230,000 to $405,000 (Table 3). This does not include salaries of the NMCP and funding agency staff involved in the evaluations. It was important for all partners to agree to the funding, either in-kind or through direct financing. This was particularly important as the impact evaluations were a large undertaking that involved multiple partners. As mentioned above, NMCPs contributed by providing staff time to serve as points of contact for the evaluation team and were involved throughout the evaluation.

**Table 3:** Sample malaria impact evaluation budget

Sample budget for conducting a malaria impact evaluation	
Activity	Cost (USD)
External (international) technical partner	145,000–175,000
Analysis of household survey data	
Analysis of additional data*	
Report writing	
Management of the evaluation process*	
Local technical partner	70,000–140,000
Compilation of data in country	
Writing background and intervention sections of the report	
Data analysis*	
Management of the evaluation process*	
Stakeholder meetings	5,000–10,000
Access and analysis of meteorological data	0–50,000
Translation services	0–15,000
Printing report (or key findings report) and dissemination meeting	10,000–15,000
Total	230,000–405,000

This is a sample budget for evaluations conducted with local and external technical partners. Salaries of national malaria control programs, funding agency staff and other reviewers working on the evaluation are not included.

* Depending on who is managing the evaluation and conducting the analyses, some costs may shift between the external and local technical partners.

Funding was necessary primarily for the analysts’ and writers’ time and this was broken into funding for in-country technical partners and external technical partners. A second cost was workshops and/or stakeholder meetings. In some cases, a payment was required for accessing data, but this was a minimal cost for evaluations based on secondary analysis of existing data sources. In some countries, funding was required for access to and analysis of the meteorological data. Planned dissemination events and printing the report or associated documents also needed to be budgeted for.

#### Human resources.

For analysts and writers, it was ideal if the evaluation team was comprised of individuals capable of analyzing large household survey data sets as well as other data sets such as those from HMIS and HDSS. In addition, there needed to be individuals on the evaluation team who were familiar with the malaria control program and could work with the NMCP to write sections on the history of malaria control in the country, policy changes, and implementation of the various malaria control interventions, and to provide context for interpreting the findings.

The core evaluation teams found that there needed to be a balance between having a large set of stakeholders involved in the evaluation and getting the evaluation done within a year. It was efficient to hire external and in-country technical partners who could devote the necessary time to the evaluations, while still consulting with the NMCP and funding agencies throughout the process.

#### Recommendations.

- A detailed budget that includes costs for human resources, stakeholder meetings, payment for data sets, subcontracts, translation, and dissemination needs to be prepared before the evaluation is implemented. This provides the basis for stakeholders to define their contributions, be it in-kind or direct financing of the evaluation.

### Independent evaluators.

One limitation of the evaluations conducted following the approach detailed in this article is that they were not conducted by truly independent evaluators.

#### Experience and lessons learned.

The evaluations described here were led by the NMCPs, funding agencies and partners, all of whom stood to gain or lose based on the results of the evaluation. The evaluation teams agreed to present the results of the evaluations whether the outcomes demonstrated impact or not; however, there still remained a potential conflict of interest, particularly because of the level of interpretation of the results that was required.

#### Recommendations.

- Encourage the evaluation team to release the evaluation findings whether they demonstrate impact or not.
- Maximize the independence of the evaluators as resources allow. Consider hiring an independent evaluator for malaria impact evaluations especially when the results are open to interpretation.

## SUMMARY

Currently, nine national-level malaria control impact evaluations have been conducted following the RBM-MERG evaluation methodology framework, with four more underway. Valuable lessons have been learned about the methods used in the evaluations^2,3,11^ and the process for conducting the evaluations. An evaluation methodology framework has been developed that can be adapted to the individual country context and a corresponding streamlined report format and operational framework as described here for conducting the evaluations have also been developed. The lessons learned from these evaluations are not unique to malaria and are applicable to other public health programs. The impact evaluations conducted thus far have relied on existing data in country. Looking forward, countries should use these evaluations to identify gaps in their data collection systems and design approaches to improve the data for monitoring the country’s malaria control efforts, with the benefit of improving the data available for future impact evaluations, including designing the evaluations prospectively. Discussions are needed to decide when follow-up impact evaluations should be conducted as many countries evaluated impact through 2010 or 2011. MoHs/NMCPs and funding agencies will need to continue to report on the impact of malaria control efforts and investments and this article and others in this supplement provide a framework for conducting impact evaluations in high burden countries. As malaria burden declines, new methods (and potentially new processes) will be needed to assess impact.
